# Phase II study of IRInotecan treatment after COmbined chemo‐immunotherapy for extensive‐stage small cell lung cancer: Protocol of IRICO study

**DOI:** 10.1111/1759-7714.15097

**Published:** 2023-09-07

**Authors:** Hiromi Tomono, Hirokazu Taniguchi, Minoru Fukuda, Takaya Ikeda, Seiji Nagashima, Kazumasa Akagi, Sawana Ono, Yasuhiro Umeyama, Midori Shimada, Hiroshi Gyotoku, Shinnosuke Takemoto, Yasushi Hisamatsu, Ryotaro Morinaga, Ryuta Tagawa, Ryosuke Ogata, Yosuke Dotsu, Hiroaki Senju, Hiroshi Soda, Katsumi Nakatomi, Fumiko Hayashi, Nanae Sugasaki, Akitoshi Kinoshita, Hiroshi Mukae

**Affiliations:** ^1^ Department of Respiratory Medicine Nagasaki University Graduate School of Biomedical Sciences Nagasaki Japan; ^2^ Department of Respiratory Medicine National Hospital Organization Nagasaki Medical Center Nagasaki Japan; ^3^ Clinical Oncology Center Nagasaki University Hospital Nagasaki Japan; ^4^ Department of Respiratory Medicine Nagasaki Prefecture Shimabara Hospital Nagasaki Japan; ^5^ Clinical Research Center Nagasaki University Hospital Nagasaki Japan; ^6^ Department of Thoracic Medical Oncology Oita Prefectural Hospital Oita Japan; ^7^ Department of Respiratory Medicine Sasebo City General Hospital Nagasaki Japan; ^8^ Department of Internal Medicine Senju Hospital Nagasaki Japan; ^9^ Department of Respiratory Medicine National Hospital Organization Ureshino Medical Center Saga Japan

**Keywords:** combined chemo‐immunotherapy, irinotecan, small cell lung cancer

## Abstract

**Introduction:**

Combined treatment using anti‐programmed death‐ligand 1 antibody (anti‐PD‐L1) and platinum‐etoposide is the current standard first‐line treatment for patients with extensive‐stage (ES) small cell lung cancer (SCLC). However, the best treatment for relapsed ES‐SCLC after the first‐line treatment remains unclear. There are some approved chemotherapeutic agents that can be used against ES‐SCLC, and treatment with irinotecan is well established as both a monotherapy and a combined therapy, in combination with platinum. Therefore, we conduct a phase II study with irinotecan in the second‐ or later‐line setting for patients with ES‐SCLC who have been previously treated with combined treatment.

**Methods:**

Our study will enroll total 30 patients who are diagnosed with ES‐SCLC and have experienced disease progression after the combined treatment. Patients will receive irinotecan on days 1, 8, and 15, which will be repeated every 4 weeks. Doses of irinotecan (100/80/60 mg/m^2^) will be determined according to the type of UGT1A1 gene polymorphism, and the treatment will be discontinued following disease progression, intolerance, withdrawal of patient consent, and based on the investigator's decision. The primary endpoint of the study is the response rate, and the secondary endpoints are overall survival, progression‐free survival, and safety.

**Discussion:**

Since the present first‐line treatment has been changed to the combined treatment, the second‐ or later‐line treatment should be re‐evaluated for patients with relapsed SCLC. Irinotecan is a major chemotherapeutic agent used for SCLC. This study demonstrates and re‐evaluates the clinical benefits of irinotecan after combined treatment with anti‐PD‐L1 and platinum‐etoposide for patients with ES‐SCLC.

**Registration details:**

This study was registered in the Japan Registry of Clinical Trials (no. jRCT s071210090) on November 4, 2021.

## Graphical abstract

Schema of IRICO study; study design, dose of irinotecan, endpoints.

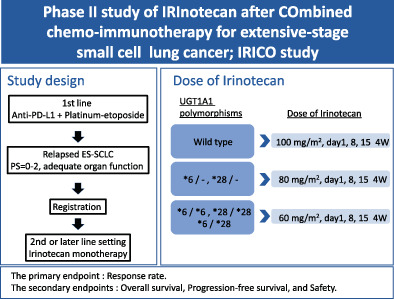

## INTRODUCTION

Small cell lung cancer (SCLC) accounts for approximately 15% of all lung cancers and tends to progress and disseminate rapidly, resulting in 70%–85% of patients being diagnosed with extensive‐stage (ES)‐SCLC, defined as the presence of metastatic lesions outside the hemithorax at the time of diagnosis.^1^, ^2^, ^3^, ^4^ Representative first‐line treatment for patients with ES‐SCLC consists of platinum and etoposide or irinotecan therapy;^5^, ^6^ however, two recent clinical trials have revealed that the addition of anti‐programmed death‐ligand 1 antibody (anti‐PD‐L1) to platinum‐etoposide therapy statistically prolonged progression‐free survival (PFS) compared to that with platinum‐etoposide therapy. Therefore, combined treatment using anti‐PD‐L1 and platinum‐etoposide is now the standard treatment in the first‐line setting for patients with ES‐SCLC.^7^, ^8^ Although treatment with anti‐PD‐L1 plus platinum‐etoposide remarkably yields a high response rate and clinical benefits, almost all patients experience relapse and acquire resistance to the treatment, suggesting the importance of the strategies in the second‐ or later‐line setting.

In cases of relapse after platinum‐etoposide treatment, the standard treatment includes a regimen identical to the initial therapy, nogitecan monotherapy,^9^, ^10^ cisplatin + etoposide + irinotecan combined therapy,^11^ and amrubicin (AMR) monotherapy.^12^, ^13^, ^14^ While these treatments demonstrate certain antitumor effects in patients with ES‐SCLC, the survival benefits are not sufficient. Furthermore, the best treatment for relapsed ES‐SCLC after first‐line combined chemo‐immunotherapy remains unclear.

Treatment with irinotecan for patients with ES‐SCLC is well established as both a monotherapy and that in combination with platinum.^5^, ^15^ Irinotecan is directly converted to its active metabolite, SN‐38, by carboxylesterase, primarily in the liver. SN‐38 is glucuronidated by UDP‐glucuronyltransferase (UGT) 1A1, a hepatic metabolic enzyme, to form an SN‐38 glucuronide conjugate (SN‐38G), which is mainly excreted in the bile. The *UGT1A1* gene is genetically polymorphic, and individuals who are heterozygous or homozygous for UGT1A1*6 and UGT1A1*28 have reduced ability to produce SN‐38G and delayed SN‐38 metabolism compared to that of the wild‐type.^16^ As a result, the incidence of grade 3 or higher neutropenia and diarrhea is higher in patients with the *UGT1A1* polymorphism. Although irinotecan therapy has been approved in many countries as a SCLC treatment, the effect of irinotecan after combined treatment with anti‐PD‐L1 and platinum‐etoposide remains unelucidated.

Our study is a phase II trial on irinotecan in the second‐ or later‐line setting after combined treatment with anti‐PD‐L1 and platinum‐etoposide against ES‐SCLC to evaluate the effect of irinotecan after treatment with anti‐PD‐L1 plus platinum‐etoposide. We will evaluate the suitability of irinotecan dosage according to the *UGT1A1* status after treatment with anti‐PD‐L1 and platinum‐etoposide.

## METHODS

### Study design

**FIGURE 1:**
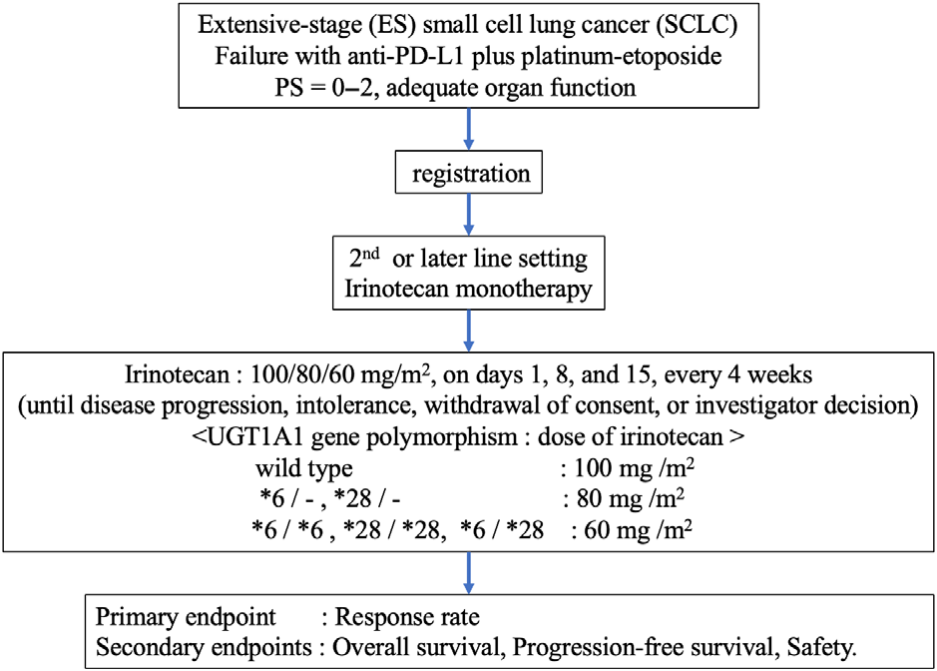
Schema of the study.

This is a multicenter, single‐arm, phase II study evaluating the efficacy and safety of investigational therapy (single‐agent irinotecan) in recurrent SCLC after treatment with anti‐PD‐L1 and platinum‐etoposide (Figure. 1). We will enroll 30 patients diagnosed with ES‐SCLC who experience disease progression after anti‐PD‐L1 and platinum‐etoposide. The overall response rate (ORR) is 44% for sensitive relapse SCLC with amrubicin 40 mg/m^2^ treatment,^13^ and 31.1% for refractory relapse SCLC with amrubicin 40 mg/m^2^ treatment.^17^ The ORR is 13% for SCLC with topotecan 1 mg/m^2^ treatment.^12^ Since 35 mg/m^2^ is often used in clinical practice and refractory relapse was included in this study, assuming an overall response rate of 25% as the threshold response rate, a target response rate of 40% was established. Based on an alpha value of 0.2 and a beta of 0.8, the estimated required number of patients was 30.

Our study is conducted in accordance with the Declaration of Helsinki and the Good Clinical Practice Guidelines and was reviewed and approved by the clinical review board of Nagasaki University (CRB21‐007‐4). All participants will provide written informed consent. The study was registered in the Japan Registry of Clinical Trials (no. jRCT 071210090) on November 4, 2021.

### Inclusion criteria

1. Presence of histologically or cytologically confirmed SCLC (combined SCLC is eligible).
2. Patients with relapse after anti‐PD‐L1 and platinum‐etoposide treatment for SCLC (both sensitive and refractory relapse cases are eligible).
3. Eastern Cooperative Oncology group performance status (PS) of 0–2.
4. Age ≥ 20 years.
5. No active malignancy (other than intraepithelial and intramucosal carcinoma equivalent lesions that has undergone potentially curative therapy) within the last 5 years prior to consent.
6. Signed written informed consent.
7. The latest laboratory results within 14 days before enrollment (the same day of the week 2 weeks before enrollment is acceptable) meet all of the following criteria: (a) neutrophil count ≥1500/μL, (b) platelet count ≥100 × 10^3^/μL, (c) total bilirubin (T‐Bil) ≤1.5 mg/dL, (d) aspartate aminotransferase (AST) ≤100 U/L, (e) alanine aminotransferase (ALT) ≤100 U/L, (f) serum creatinine (Cr) ≤1.5 mg/dL, (g) SpO_2_ on room air ≥92%.

### Exclusion criteria

1. Interstitial pneumonia that can be detected using chest radiographs.
2. Chest radiation treatment within 28 days.
3. Superior vena cava syndrome that requires urgent radiation therapy.
4. Clinically serious cardiac disease such as angina pectoris, uncontrolled heart failure, or myocardial infarction within 3 months.
5. Uncontrolled diabetes mellitus.
6. Uncontrolled hypertension.
7. Active systemic infection that requires treatment.
8. Pregnant, or possibly pregnant, postpartum in 28 days, breastfeeding.
9. Fever of ≥38.0°C.
10. Mental disorder or symptoms interfering with daily life and inappropriate for enrollment.
11. Receiving continuous systemic administration (oral or intravenous) of steroids or immunosuppressive drugs equivalent to 11 mg/day or more of prednisolone.
12. Positive for hepatitis B surface antigen.
13. Positive for human immunodeficiency virus antibody (not necessarily examined).
14. Other inappropriate complications.

### Interventions

Patients will receive irinotecan on days 1, 8, and 15, which will be repeated every 4 weeks. Doses of irinotecan will be determined according to the type of *UGT1A1* gene polymorphism (wild type: 100 mg/m^2^, *6/− or *28/−: 80 mg/m^2^, *6/*6, *28/*28 or *6/*28: 60 mg/m^2^), and treatment will be continued until disease progression, intolerance is detected, or the patient withdraws consent, and based on the investigator's decision.

### Endpoints

The primary endpoint of the present study is the response rate, and the secondary endpoints are overall survival (OS), progression‐free survival (PFS), and safety. Efficacy will be assessed according to the Response Evaluation Criteria in Solid Tumors version 1.1. PFS is defined as the time from registration to disease progression or death from any cause, whereas OS is defined as the time from registration to the last day confirmed to be alive or dead from any cause. The Kaplan–Meier method will be used to estimate PFS and OS. Survival analysis will be conducted at the end of the follow‐up period.

## DISCUSSION

To our knowledge, this is the first prospective study to evaluate the effect of irinotecan after combined therapy of anti‐PD‐L1 and platinum‐etoposide. Our study will provide a convincing option of irinotecan therapy after anti‐PD‐L1 and platinum‐etoposide therapy in treating SCLC if the primary endpoint is achieved.

In our previous study, the sequence of chemotherapy following nivolumab in non‐small cell lung cancer enhanced the antitumor activity of cytotoxic chemotherapy.^18^ In addition, a recent retrospective study revealed that the efficacy of AMR therapy after chemo‐immunotherapy in patients with SCLC showed an overall response rate of 47% (95% confidence interval [CI]: 30%–64%), and the disease‐control rate of 73% (95% CI: 56%–86%),^19^ indicating that single agent of AMR is a useful treatment option after chemo‐immunotherapy. Irinotecan is a topoisomerase I inhibitor and a major chemotherapeutic agent and has been used for treating SCLC for a long time. The response rates of 47% in 16 previously treated SCLC patients^15^ and 79% in untreated patients with advanced SCLC in combination with cisplatin^20^ have been reported. Furthermore, sequential administration of topoisomerase I inhibitors (irinotecan and nogitecan) and topoisomerase II inhibitors (etoposide and AMR) have been suggested to show clinical benefits.^21^ The current standard first‐line treatment includes anti‐PD‐L1, cisplatin, topoisomerase II inhibitor (etoposide); therefore, this study may provide valuable results for treating patients with SCLC.

Consistent with the significant associations between the UGT1A1*6 genotype and irinotecan‐induced toxicities in Asian populations, the Japanese Pharmaceuticals and Medical Devices Agency recommends screening patients for UGT1A1*6 and *28 polymorphisms. The US Food and Drug Administration also recommends a reduction in the irinotecan starting dose in patients who are homozygous for UGT1A1*28.^16^ Therefore, we aim to adjust the irinotecan dose according to the UGT1A1 type and demonstrate that irinotecan can be safely administered even in patients with UGT1A1*6 and UGT1A1*28 genotypes.

In conclusion, our study will demonstrate and re‐evaluate the efficacy and safety of irinotecan after combined treatment with anti‐PD‐L1 and platinum‐etoposide for patients with ES‐SCLC.

## AUTHOR CONTRIBUTIONS

All authors had full access to the study protocol and took responsibility for its integrity. Conceptualization, Minoru Fukuda; Investigation, Hiromi Tomono. Hirokazu Taniguchi, Minoru Fukuda, Takaya Ikeda, Seiji Nagashima, Kazumasa Akagi, Sawana Ono, Yasuhiro Umeyama, Midori Shimada, Hiroshi Gyotoku, Shinnosuke Takemoto, Yasushi Hisamatsu, Ryotaro Morinaga, Ryuta Tagawa, Ryosuke Ogata, Yosuke Dotsu, Hiroaki Senju, Hiroshi Soda. Katsumi Nakatomi, Fumiko Hayashi, Nanae Sugasaki, Akitoshi Kinoshita; Methodology, Minoru Fukuda; Project Administration, Hirokazu Taniguchi and Minoru Fukuda; Supervision, Hiroshi Mukae; Visualization, Hiromi Tomono. Writing—original draft preparation, Hiromi Tomono and Hirokazu Taniguchi. Writing—review & editing, Hirokazu Taniguchi, Minoru Fukuda, Takaya Ikeda, Seiji Nagashima, Kazumasa Akagi, Sawana Ono, Yasuhiro Umeyama, Midori Shimada, Hiroshi Gyotoku, Shinnosuke Takemoto, Yasushi Hisamatsu, Ryotaro Morinaga, Ryuta Tagawa, Ryosuke Ogata, Yosuke Dotsu, Hiroaki Senju, Hiroshi Soda, Katsumi Nakatomi, Fumiko Hayashi, Nanae Sugasaki, Akitoshi Kinoshita, and Hiroshi Mukae.

## CONFLICT OF INTEREST STATEMENT

All the authors declare no potential conflicts of interest related to this work.
